# Presbycusis Disrupts Spontaneous Activity Revealed by Resting-State Functional MRI

**DOI:** 10.3389/fnbeh.2018.00044

**Published:** 2018-03-13

**Authors:** Yu-Chen Chen, Huiyou Chen, Liang Jiang, Fan Bo, Jin-Jing Xu, Cun-Nan Mao, Richard Salvi, Xindao Yin, Guangming Lu, Jian-Ping Gu

**Affiliations:** ^1^Department of Medical Imaging, Jinling Hospital, School of Medicine, Nanjing University, Nanjing, China; ^2^Department of Radiology, Nanjing First Hospital, Nanjing Medical University, Nanjing, China; ^3^Department of Otolaryngology, Nanjing First Hospital, Nanjing Medical University, Nanjing, China; ^4^Center for Hearing and Deafness, University at Buffalo, State University of New York, Buffalo, NY, United States

**Keywords:** presbycusis, ALFF, ReHo, resting-state fMRI, spontaneous activity

## Abstract

**Purpose**: Presbycusis, age-related hearing loss, is believed to involve neural changes in the central nervous system, which is associated with an increased risk of cognitive impairment. The goal of this study was to determine if presbycusis disrupted spontaneous neural activity in specific brain areas involved in auditory processing, attention and cognitive function using resting-state functional magnetic resonance imaging (fMRI) approach.

**Methods**: Hearing and resting-state fMRI measurements were obtained from 22 presbycusis patients and 23 age-, sex- and education-matched healthy controls. To identify changes in spontaneous neural activity associated with age-related hearing loss, we compared the amplitude of low-frequency fluctuations (ALFF) and regional homogeneity (ReHo) of fMRI signals in presbycusis patients vs. controls and then determined if these changes were linked to clinical measures of presbycusis.

**Results**: Compared with healthy controls, presbycusis patients manifested decreased spontaneous activity mainly in the superior temporal gyrus (STG), parahippocampal gyrus (PHG), precuneus and inferior parietal lobule (IPL) as well as increased neural activity in the middle frontal gyrus (MFG), cuneus and postcentral gyrus (PoCG). A significant negative correlation was observed between ALFF/ReHo activity in the STG and average hearing thresholds in presbycusis patients. Increased ALFF/ReHo activity in the MFG was positively correlated with impaired Trail-Making Test B (TMT-B) scores, indicative of impaired cognitive function involving the frontal lobe.

**Conclusions**: Presbycusis patients have disrupted spontaneous neural activity reflected by ALFF and ReHo measurements in several brain regions; these changes are associated with specific cognitive performance and speech/language processing. These findings mainly emphasize the crucial role of aberrant resting-state ALFF/ReHo patterns in presbycusis patients and will lead to a better understanding of the neuropathological mechanisms underlying presbycusis.

## Introduction

Presbycusis, or age-related hearing loss, is the most common hearing disorder and a major cause of chronic disability in the elderly, which can lead to social isolation, communication, language and speech processing problems and depression (Davis, 1991; Gates and Mills, 2005; Dubno et al., 2008; Sprinzl and Riechelmann, 2010; Rosenhall et al., 2011; Panza et al., 2015). Age-related hearing loss not only disrupts inputs to the primary auditory cortex and receptive language areas, but also language association areas that assign meaning to words and other meaningful sounds (Salvi et al., 2002; Ardila et al., 2016). Almost two-thirds of elderly Americans have age-related hearing loss consistent with comparable trends around the globe (Lin et al., 2011b; Feder et al., 2015). While age-related hearing loss results in large part from the loss of sensory hair cell and neurons in the cochlea, the sensory organ for hearing, there is growing awareness that presbycusis is also associated with structural and functional changes in the central auditory pathway as well as other regions in the central nervous system (Kazee et al., 1995; Spongr et al., 1997; Salvi et al., 2002; Ouda et al., 2015). In the auditory midbrain of rats with age-related hearing loss, sound evoked firing rates were reduced and tuning was broader (Palombi and Caspary, 1996). However, in primary auditory cortex of rats and nonhuman primates with age-related hearing loss, sound-evoked and spontaneous activity were increased, but temporal processing and response selectivity were degraded (Hughes et al., 2010; Chi-Wing and Recanzone, 2017). Changes in spontaneous rate are not uniform across the auditory cortex; while those in primary auditory cortex decrease following noise-induced hearing loss, those in the anterior auditory field did not change while those in secondary auditory cortex decreased (Kimura and Eggermont, 1999). Age-related hearing loss also disrupts the tonotopic organization of the central auditory pathway (Willott, 1991). Consistent with animal studies, sound-evoked functional magnetic resonance imaging (fMRI) response in auditory cortex of subjects with mild or more severe presbycusis were greater than young controls and these differences were more pronounced in the right temporal lobe of the elderly (Profant et al., 2015). Others have reported decreased activation of the contralateral auditory cortex and a reduced right-ear advantage with aging and presbycusis (Chen et al., 2016). Some fMRI studies have reported that age-related hearing loss increases the functional connectivity between the auditory cortex and right motion sensitive visual area during task and resting-state conditions (Puschmann and Thiel, 2017).

Data from large population-based longitudinal studies have shown that presbycusis is associated with an increased risk of cognitive impairment and incident dementia, which primarily manifests as declining executive function (Lin et al., 2011a, b; Gurgel et al., 2014), verbal memory (Tay et al., 2006; Lin et al., 2011a), episodic and semantic long-term memory (Ronnberg et al., 2011) and psychomotor processing (Lin et al., 2011b). However, the pathophysiological mechanisms underlying presbycusis and its association with cognitive impairments remain poorly understood.

Neuroimaging technique has been applied to investigate the anatomical and functional alterations in the brains of patients with presbycusis (Mudar and Husain, 2016). Gray matter (GM) atrophy and white matter (WM) lesions, common structural abnormalities observed in previous studies, are modestly linked with presbycusis-related cognitive decline (Peelle et al., 2011; Eckert et al., 2012; Lin et al., 2014; Profant et al., 2014; Ma et al., 2016). Gamma-aminobutyric acid (GABA) levels in the auditory cortex, an important inhibitory neurotransmitter that can be assessed by magnetic resonance spectroscopy (MRS), were negatively correlated with the degree of age-related hearing loss (Gao et al., 2015). Nonetheless, little is known about the complex neurophysiological changes in the central nervous system likely to be associated with presbycusis. Neural abnormalities have been detected in populations at risk for developing cognitive impairment (Machulda et al., 2011). Therefore, measures of neural activity could conceivably be used to detect and track the possible effects of presbycusis on brain function.

Most prior studies have used fMRI to examine the effects of hearing loss on the brain activity during auditory tasks in presbycusis patients (Peelle et al., 2011; Husain et al., 2014; Profant et al., 2015; Chen et al., 2016; Puschmann and Thiel, 2017). Resting-state fMRI (rs-fMRI) is a powerful tool for evaluating spontaneous neural activity (Mantini et al., 2007) and rs-fMRI has been used to investigate brain functional connectivity alterations in the default mode network and dorsal attention network in middle-aged participants with hearing loss compared to controls (Husain et al., 2014). A recent rs-fMRI study found that hearing loss modulated cross-modal functional connectivity between visual and auditory sensory cortices in presbycusis (Puschmann and Thiel, 2017), but did not evaluate other measures of resting-state neural activity. As the effects of hearing loss on the brain may be global, a whole-brain analysis of neural function would likely identify other central processing deficits linked to presbycusis.

Amplitude of low-frequency fluctuation (ALFF) and regional homogeneity (ReHo) are two important data-driven algorithms for analyzing global rs-fMRI signals. ALFF measures the amplitude of very low-frequency oscillations of the BOLD signal at the single-voxel level (Zang et al., 2007) while ReHo analyses the neural synchronization of a given voxel with its adjacent voxels, i.e., local neural synchrony (Zang et al., 2004). ReHo may be more sensitive at detecting regional brain abnormalities than ALFF. On the other hand, ALFF may be more useful than ReHo for investigating global changes in spontaneous neural activity (An et al., 2013; Zhang et al., 2015). Thus, the combination of ALFF and ReHo may provide a more comprehensive pathophysiological assessment of human brain dysfunction than either method alone. Therefore, we took advantage of the global and local analytical power of ALFF and ReHo to identify aberrant spontaneous neural activity in presbycusis patients. Based on prior human and animal studies noted above, we hypothesized that aberrant ALFF and ReHo values would be detected in specific brain regions involved in complex auditory processing (e.g., speech or language comprehension), attention and cognitive function.

## Materials and Methods

### Subjects

All the subjects provided written informed consent before their participation in the study protocol, which was approved by the Research Ethics Committee of the Nanjing Medical University. A total of 22 presbycusis patients with disease duration of 3–10 years were recruited for this study at the Department of Otolaryngology of Nanjing First Hospital. Hearing thresholds were determined by pure tone audiometry (PTA) at the frequencies of 0.25, 0.5, 1, 2, 4 and 8 kHz. The criteria for the estimation of the disease duration is the results of average PTA. Average PTA values of ≤25 dB hearing level (HL) were regarded as within the limit for normal hearing thresholds (Lin et al., 2011b). For the presbycusis group, audiometric thresholds inclusion criteria were average PTA >25 dB HL in the better hearing ear and age ≧60 years. Twenty-three age, sex, and education-matched healthy subjects with no hearing loss (average PTA ≦25 dB in the better-hearing ear) were recruited through community health screenings or newspaper advertisements. All subjects were right-handed and completed at least 8 years of education. Tympanometry was performed with a Madsen Electronics Zodiac 901 Middle Ear Analyzer (GN Otometrics) to confirm normal middle-ear function. None of the participants were excluded from the study because of excessive head movement during MR scanning. Exclusion criteria were ear diseases that affect hearing thresholds and sensorineural hearing loss other than presbycusis. Participants were excluded from the study if they suffered from tinnitus, hyperacusis, Meniere’s diseases, or had a past history of otologic surgery, ototoxic drug therapy, noise exposure, or hearing aid use severe smoking, stroke, alcoholism, brain injury, Parkinson’s disease, Alzheimer’s disease (AD), epilepsy, major depression, neurological or psychiatric disorders that could affect cognitive function, major medical illness (e.g., anemia, thyroid dysfunction and cancer), MRI contraindications, or severe visual loss. The characteristics of the presbycusis patients and healthy subjects are summarized in Table 1. All the participants were randomly assigned under double-blind conditions for further data analysis.

**Table 1:** Demographics, clinical and cognitive characteristics of the presbycusis patients and healthy controls.

	Presbycusis patients (*n* = 22)	Healthy controls (*n* = 23)	*p* value
Age (years)	63.59 ± 2.38	64.74 ± 2.65	0.134
Gender (male: female)	10:12	11:12	0.873
Education levels (years)	10.64 ± 1.89	10.35 ± 1.53	0.575
Disease duration (years)	4.80 ± 2.17	–	–
PTA (dB HL)	34.54 ± 4.63	14.82 ± 1.73	<0.001
FD value	0.21 ± 0.06	0.20 ± 0.07	0.904
**Cognitive performance**		
MMSE	28.77 ± 1.23	28.74 ± 1.36	0.931
MoCA	25.59 ± 1.74	25.65 ± 1.82	0.909
AVLT	34.14 ± 8.84	33.30 ± 7.12	0.729
AVLT-delayed recall	6.82 ± 2.77	6.39 ± 1.92	0.550
CFT	34.52 ± 1.56	34.24 ± 1.90	0.588
CFT-delayed recall	16.64 ± 2.87	16.48 ± 3.19	0.862
DST	11.23 ± 1.48	11.87 ± 2.28	0.271
TMT-A	71.77 ± 21.23	68.70 ± 21.27	0.630
TMT-B	197.45 ± 61.30	158.00 ± 52.41	0.025*
CDT	3.50 ± 0.60	3.60 ± 0.50	0.511
VFT	13.64 ± 4.65	14.07 ± 3.43	0.726
SDS	42.05 ± 9.91	37.00 ± 7.56	0.061
SAS	38.50 ± 6.15	36.57 ± 6.22	0.300

Data are represented as Mean ± SD. The PTA from both ears was averaged. *P value < 0.05. PTA, puretone audiometry; FD, framewise displacement; MMSE, Mini Mental State Exam; MoCA, Montreal Cognitive Assessment; AVLT, Auditory Verbal Learning Test; CFT, Complex Figure Test; DST, Digit Span Test; TMT-A, Trail Making Test-Part A; TMT-B, Trail Making Test-Part B; CDT, Clock Drawing Test; VFT, Verbal Fluency Test; SDS, Self-Rating Depression Scale; SAS, Self-Rating Anxiety Scale.

### Neuropsychological Assessment

All participants underwent a battery of neuropsychological tests that covered relevant cognitive domains. The neuropsychological status of the participants was established using the Mini Mental State Exam (MMSE; Galea and Woodward, 2005), Montreal Cognitive Assessment (MoCA; Nasreddine et al., 2005), Auditory Verbal Learning Test (AVLT; Schmidt, 1996), Complex Figure Test (CFT; Shin et al., 2006), Digit Span Test (DST; Hale et al., 2002), Trail-Making Test (TMT) A and B (Bowie and Harvey, 2006), Clock-Drawing Test (CDT; Samton et al., 2005) and Verbal Fluency Test (VFT; Brucki and Rocha, 2004). The tests provided an assessment reflecting the general cognitive function, episodic verbal and visual memory, semantic memory, attention, psychomotor speed, executive function and visuospatial skills. Depression and anxiety status were assessed according to the Self-Rating Depression Scale (SDS) and Self-Rating Anxiety Scale (SAS; Zung, 1971, 1986). It took approximately 60 min for each individual to complete all the tests in a fixed order.

### MRI Acquisition

MRI data were acquired at using a 3.0 T MRI scanner (Ingenia, Philips Medical Systems, Netherlands) with an 8-channel receiver array head coil. Head motion and scanner noise were reduced using foam padding and earplugs. The earplugs (Hearos Ultimate Softness Series, USA) were used to attenuate scanner noise by approximately 32 dB based on the manufacture’s data sheet. Subjects were instructed to lie quietly with their eyes closed without falling asleep, not think of anything in particular, and avoid any head motion during the scan. Functional images were obtained axially using a gradient echo-planar imaging sequence as follows: repetition time (TR) = 2000 ms; echo time (TE) = 30 ms; slices = 36; thickness = 4 mm; gap = 0 mm; flip angle (FA) = 90°; field of view (FOV) = 240 mm × 240 mm; acquisition matrix = 64 × 64; voxel size = 3.75 × 3.75 × 4.0 mm^3^. The fMRI sequence took 8 min and 8 s. Structural images were acquired with a three-dimensional turbo fast echo (3D-TFE) T1WI sequence with high resolution as follows: TR/TE = 8.2/3.8 ms; slices = 170; thickness = 1 mm; gap = 0 mm; FA = 8°; acquisition matrix = 256 × 256; FOV = 256 mm × 256 mm. The structural sequence took 5 min and 29 s.

### Functional Data Preprocessing

Functional data analyses were conducted using Data Processing Assistant for Resting-State fMRI (DPARSF) programs (Yan et al., 2016) based on statistical parametric mapping (SPM8)^1^ and resting-state fMRI data analyses toolkits (REST)^2^. A total of 240 volumes were scanned, and the first 10 volumes were discarded to allow for signal equilibrium of the initial magnetic resonance signals and adaptation of the subjects to scanner. The remaining 230 consecutive volumes were used for data analysis. Afterwards, the following procedures were carried out as follows: slice-timing adjustment, realignment for head-motion correction, spatial normalization to the Montreal Neurological Institute (MNI) template (resampling voxel size = 3 × 3 × 3 mm^3^) as well as linear trending and band-pass filtering (0.01–0.08 Hz). Any subjects with a head motion >2.0 mm translation or 2.0° rotation in any direction were excluded.

### ALFF and ReHo Analyses

For ALFF analysis, the images were first smoothed with a Gaussian kernel of 6 mm full-width at half maximum (FWHM). Next, the time courses were first transformed to the frequency domain using a Fast Fourier Transform algorithm. The square root was then computed at each frequency of the power spectrum. ALFF values were obtained after calculation at each frequency of the power spectrum across 0.01–0.08 Hz at each voxel. For standardization purposes, the ALFF of each voxel was divided using the global mean ALFF value. ALFF was finally calculated using the REST software through the procedure described in previous studies (Zang et al., 2007).

For ReHo analysis, the images were calculated for Kendall’s coefficient of concordance of the time series of a given voxel with its 26 nearest neighboring voxels. The ReHo maps were then spatially smoothed with a Gaussian kernel of 6 mm. The ReHo value of each voxel was standardized by dividing the raw value by the global mean ReHo value, which was obtained with a similar calculation used to determine the global man ALFF value. ReHo was also calculated using the REST software through the procedure described in previous studies (Zang et al., 2004).

### Structural Analysis

Structural images were processed using the VBM toolbox software in SPM8^1^. Briefly, the structural images were normalized and segmented into GM, WM and cerebrospinal fluid (CSF) using the unified segmentation model in SPM8. Brain parenchyma volume was calculated as the sum of GM and WM volumes. The GM images were spatially smoothed using a Gaussian kernel of 8 mm FWHM. The voxel-wise GM volume was used in the following statistical analysis as covariates for ALFF and ReHo calculations.

### Statistical Analysis

Demographic and clinical variables and cognitive performance scores were compared between the two groups using SPSS software (version 20.0; SPSS, Inc., Chicago, IL, USA). An independent two-sample *t* test was used for continuous variables, and a *χ*^2^ test was used for proportions. *p* < 0.05 were considered to be statistically significant.

#### Within-Group Analysis

For within-group whole brain ALFF and ReHo patterns, one-sample *t*-tests were performed on the individual ALFF and ReHo maps in a voxel-wise manner for patient and healthy control group. Significant thresholds were corrected using false discovery rate (FDR) criterion and the significance set at *p* < 0.001. The group-level ALFF and ReHo maps were then visualized with the REST Slice Viewer in REST software.

#### Between-Group Analysis

To investigate between-group differences of ALFF and ReHo values, two-sample *t*-tests were performed with REST software (within a GM mask). Age, sex, education levels and mean hearing thresholds were imported as covariates. To exclude potential effects of GM volume differences, the voxel-wise GM volume maps were also obtained as covariates. Multiple comparison correction was performed using FDR criterion and the significance set at *p* < 0.001 according to the suggestion from the prior study (Eklund et al., 2016). The results were visualized using the REST Slice Viewer.

#### Correlation Analysis

To identify associations between regional ALFF and ReHo abnormalities, a bivariate correlation was performed between these two measurements. The average ALFF and ReHo values of brain regions with significant differences were individually extracted and correlated with one another.

To investigate the relationship among ALFF/ReHo values of the peak voxels, neuropsychological performances and HLs, Pearson’s correlative analyses were performed using SPSS software. Partial correlations were calculated using the same covariates as those controlled in the two-sample *t*-tests. *p* < 0.05 was considered to be statistically significant. Bonferroni correction was used for multiple comparisons in the correlation analyses.

Since micromovements from volume to volume can influence the spontaneous neuronal activity (Power et al., 2012), framewise displacement (FD) values were computed for each subject to reflect the temporal derivative of the movement parameters. No subjects had FD > 0.5 mm on greater than 35 volumes in this study. No significant difference was found in the mean FD values between presbycusis patients and controls (Table 1).

## Results

### Demographic and Neuropsychological Characteristics

**Figure 1:**
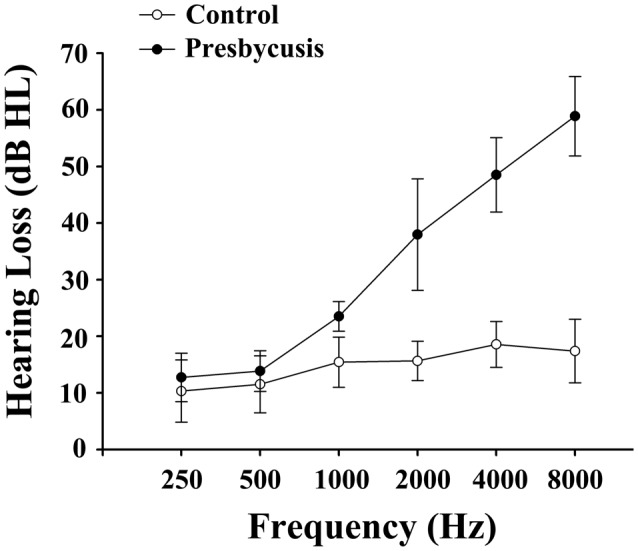
Average hearing thresholds of the presbycusis and control groups. The hearing thresholds were significantly higher in presbycusis patients than in healthy controls (*p* < 0.001, 1–8 kHz). In the presbycusis group, the mean hearing thresholds were >20 dB hearing level (HL) at 1 kHz and reached 38.0 dB HL at 2 kHz; 48.5 dB HL at 4 kHz and 58.9 dB HL at 8 kHz. Data are presented as mean ± SD.

Table 1 summarizes the demographic measures and neuropsychological test results of presbycusis patients and healthy controls. Since no significant differences in PTA between the left and right ears were observed in the presbycusis group and the control group, the thresholds of both ears were averaged and are presented for each group. The PTA was significantly higher in presbycusis patients than in healthy controls (*p* < 0.001, 1–8 kHz). In the presbycusis group, the mean hearing thresholds were >20 dB HL at 1 kHz and reached 38.0 dB HL at 2 kHz; 48.5 dB HL at 4 kHz and 58.9 dB HL at 8 kHz (Figure 1). In the control group, the mean hearing thresholds were <20 dB HL at all frequencies. All patients had a type-A curve on tympanometry, which indicated normal middle-ear function. On cognitive tests, presbycusis patients performed significantly poorer on Trail-Making Test B (TMT-B; *p* < 0.05). On the other neuropsychological tests, presbycusis patients have slightly lower scores than controls, but the differences were not significantly different.

### Structural Results

Table 2 shows no significant difference in GM, WM or brain parenchyma volume in patients with presbycusis compared to healthy controls. After Monte Carlo simulation correction, no suprathreshold voxel-wise difference in the GM and WM volume between the presbycusis patients and healthy controls was observed. None of the participants in this study were excluded due to severe atrophy.

**Table 2:** Comparisons of the brain volumes between tinnitus patients and healthy controls.

	Presbycusis patients (*n* = 22)	Healthy controls (*n* = 23)	*p* value
Gray matter	564.0 ± 24.4	571.2 ± 20.8	0.294
White matter	526.8 ± 16.5	529.0 ± 25.6	0.737
Brain parenchyma	1090.8 ± 34.4	1100.2 ± 40.6	0.410

*Data are expressed as Mean ± SD*.

### ALFF and ReHo Analyses

**Figure 2:**
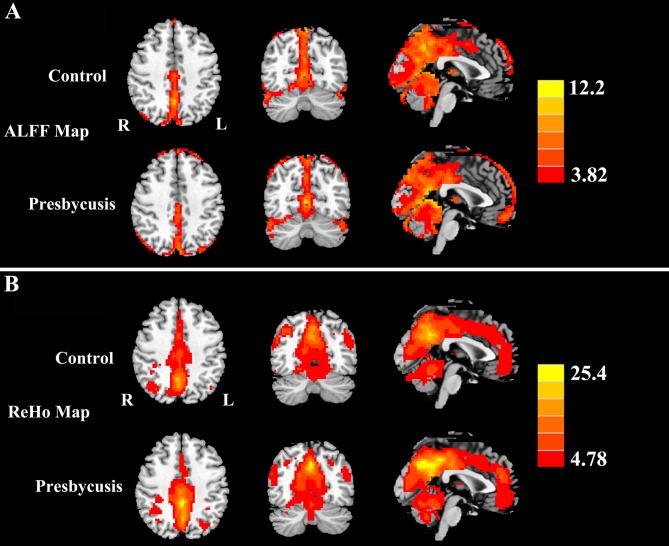
**(A)** One-sample *t*-test results of amplitude of low-frequency fluctuations (ALFF) maps in presbycusis and controls groups (*p* < 0.001 corrected by false discovery rate, FDR). **(B)** One-sample *t*-test results of regional homogeneity (ReHo) maps in presbycusis and controls groups (*p* < 0.001 corrected by FDR). The ALFF and ReHo values were significantly higher than the global means values mainly in the superior temporal gyrus (STG), precuneus, posterior cingulate cortex (PCC), cuneus and medial prefrontal gyrus. Left side corresponds to the right hemisphere.

**Figure 3:**
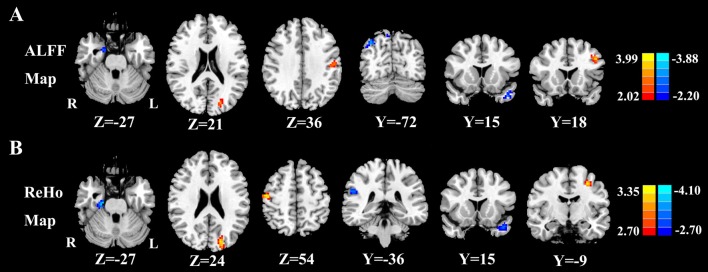
**(A)** Compared with healthy controls, presbycusis patients showed significantly decreased ALFF values in the left STG, right parahippocampal gyrus (PHG) and right precuneus, but increased ALFF values in the left middle frontal gyrus (MFG), left cuneus and left postcentral gyrus (PoCG; *p* < 0.001 corrected by FDR). **(B)** Compared with healthy controls, presbycusis patients had significantly decreased ReHo values in the left STG, right PHG and right inferior parietal lobule (IPL), but increased ReHo values in the left MFG, left cuneus and right PoCG. Left side corresponds to the right hemisphere.

Figure 2 shows the standardized ALFF (Figure 2A) and ReHo (Figure 2B) heat map for the control group and presbycusis group. ALFF and ReHo values were significantly higher than the global means values mainly in the superior temporal gyrus (STG), precuneus, posterior cingulate cortex (PCC), cuneus and medial prefrontal gyrus. Compared with healthy controls, presbycusis patients had significantly decreased ALFF values in the left STG, right parahippocampal gyrus (PHG) and right precuneus, but increased ALFF values in the left middle frontal gyrus (MFG), left cuneus and left postcentral gyrus (PoCG; Figure 3A, Table 3). Compared to healthy controls, ReHo values in presbycusis patients were significantly decreased in the left STG, right PHG and right inferior parietal lobule (IPL), but significantly increased in the left MFG, left cuneus and right PoCG (Figure 3B, Table 3).

**Table 3:** Regions showing significant differences in amplitude of low-frequency fluctuations (ALFF) and regional homogeneity (ReHo) values between presbycusis patients and healthy controls.

Brain regions	BA	Peak MNI coordinates *x, y, z* (mm)	Peak *T* value	Voxels
**ALFF differences**				
L STG	38	−42, 15, −33	−3.1602	43
R PHG	36	21, −3, −27	−2.9921	47
R Precuneus	7	36, −72, 45	−3.3427	116
L MFG	9	−45, 18, 33	3.7698	149
L Cuneus	19	−21, −81, 21	3.3929	53
L PoCG	3	−54, −15, 36	3.1611	68
**ReHo differences**				
L STG	38	−45, 15, −27	−3.0727	60
R PHG	36	21, −18, −27	−3.8228	132
R IPL	40	54, −36, 27	−3.1952	55
L MFG	9	−30, −9, 48	3.3943	70
L Cuneus	19	−18, −84, 24	3.4866	110
R PoCG	3	51, −15, 54	3.4056	57

*The threshold was set at a *p* < 0.001 (false discovery rate, FDR corrected). BA, Brodmann’s area; MNI, Montreal Neurological Institute; STG, superior temporal gyrus; PHG, parahippocampal gyrus; MFG, middle frontal gyrus; PoCG, postcentral gyrus; IPL, inferior parietal lobule; L, left; R, right*.

### Correlation Analyses

**Figure 4:**
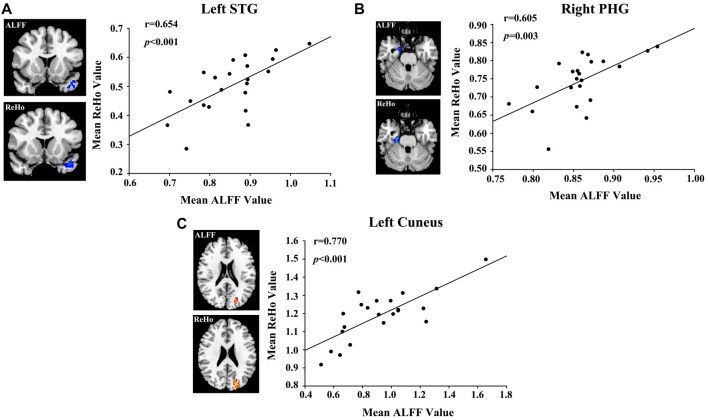
Correlations between the ALFF and ReHo values in **(A)** left STG (*r* = 0.654, *p* < 0.001), **(B)** right PHG (*r* = 0.605, *p* = 0.003) and **(C)** left cuneus (*r* = 0.770, *p* < 0.001).

**Figure 5:**
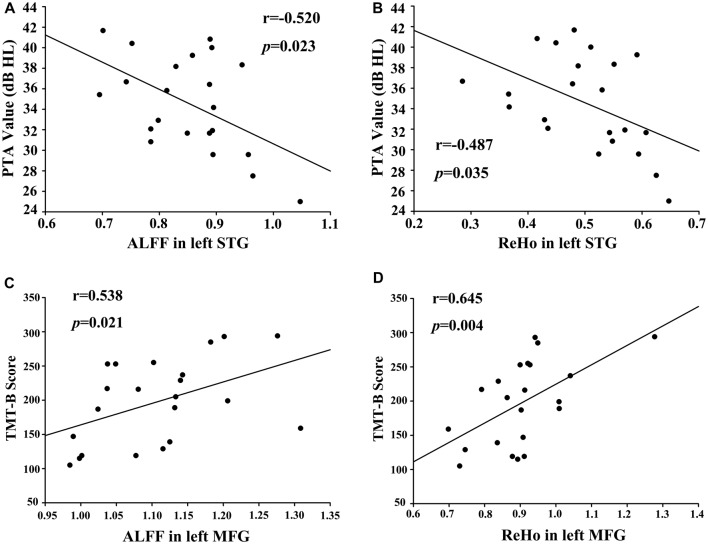
**(A)** Correlations between the mean pure tone audiometry (PTA) values and decreased ALFF values in left STG (*r* = −0.520, *p* = 0.023). **(B)** Correlations between mean PTA values and decreased ReHo values in left STG (*r* = −0.487, *p* = 0.035). **(C)** Correlations between the Trail-Making Test B (TMT-B) scores and increased ALFF in left MFG (*r* = 0.538, *p* = 0.021). **(D)** Correlations between TMT-B scores and increased ReHo in left MFG (*r* = 0.645, *p* = 0.004).

A bivariate correlation analysis indicated that regional ALFF and ReHo values extracted from the left STG (*r* = 0.654, *p* < 0.001), right PHG (*r* = 0.605, *p* = 0.003), and left cuneus (*r* = 0.770, *p* < 0.001) were strongly associated with each other in the presbycusis patients (Figure 4). After correcting for age, sex, education and GM volume, the ALFF (Figure 5A) and ReHo (Figure 5B) values in the left STG were negatively correlated with the mean PTA values in the presbycusis patients (*r* = −0.520, *p* = 0.023; *r* = −0.487, *p* = 0.035, respectively). Among the cognitive measures, TMT-B scores were positively correlated with the ALFF (Figure 5C) and ReHo (Figure 5D) values in the left MFG (*r* = 0.538, *p* = 0.021; *r* = 0.645, *p* = 0.004, respectively), corrected for age, sex, education, GM volume and mean PTA values. The other significant decreases or increases in ALFF/ReHo values were independent (i.e., no significant correlations) of hearing loss or any other cognitive tests.

## Discussions

To our knowledge, this is the first fMRI study to use both ALFF and ReHo to determine if spontaneous neural activity is disrupted in presbycusis patients and to ascertain if the disruptions in spontaneous activity are correlated with executive or cognitive decline. Increased or decreased neuronal changes were detected primarily in the left STG (BA 38), prefrontal cortex and portions of the DMN (e.g., precuneus and IPL). Moreover, a significant correlation was found between ALFF and ReHo values in the left STG (BA 38) and average PTA values, specifically ALFF and ReHo values decreased with increasing hearing loss in presbycusis patients. The left STG plays an important role in speech comprehension (Giraud et al., 2004) and auditory temporal processing, a psychoacoustic property that is disrupted by presbycusis and high frequency hearing loss (Salvi and Arehole, 1985; Zatorre and Belin, 2001; Gordon-Salant, 2005; Ozmeral et al., 2016). In addition, ALFF and ReHo activity in the left MFG increased significantly with decline in executive function as reflected in increasing TMT-B test scores, which implicates executive dysfunction as a putative mechanism involved in presbycusis.

ALFF and ReHo have been widely used to explore the pathogenesis of various neuropsychiatric disorders (Zang et al., 2004, 2007; Wang et al., 2011; An et al., 2013; Zhang et al., 2015). ALFF measures the intensity of spontaneous low-frequency oscillations (Zang et al., 2007) whereas ReHo reflects the local neural coherence of the underlying spontaneous neural activity (Zang et al., 2004). In the current study, ALFF and ReHo values were significantly higher than the global means values mainly in the DMN regions. Previous studies using positron emission tomography (PET) have confirmed that the metabolism of the DMN is higher than the global means values in the whole brain, which is also observed in our study utilizing the ALFF and ReHo methods, reflecting that neural activity in the DMN is most active at rest as distinguished from regions that are transiently activated in support of varying goal-directed activities (Raichle et al., 2001; Raichle and Snyder, 2007). In presbycusis patients, the increase in ALFF intensity was strongly correlated with increased ReHo coherence values in three regions, the left STG, left cuneus and right PHG. The simultaneous increase of both amplitude and coherence may reflect a more severe or complex dysfunction in these three regions. Surprisingly, the changes in the PoCG were different for ALFF and ReHo in our results, which could probably be due to the different mathematical algorithms between these two methods (Zang et al., 2004, 2007). Moreover, An et al. also suggested that there existed several abnormal brain regions that did not overlap in the same lateralization although the same brain area was detected by using ALFF and ReHo algorithms separately (An et al., 2013).

Elderly patients with presbycusis and other forms of hearing loss often suffer from tinnitus (Baracca et al., 2011). One major finding of this study was that presbycusis patients showed decreased ALFF and ReHo values in the left STG (BA 38), which were correlated with the mean PTA values. Interestingly, in profoundly deaf patients with tinnitus, activation of their cochlear implant temporarily suppresses tinnitus (residual inhibition) and activates BA 38. One interpretation of these results is that cochlear deafness suppresses spontaneous activity in BA 38, creating conditions that may allow the phantom sound of tinnitus to emerge, i.e., a certain level of spontaneous activity in BA 38 may be needed to suppress phantom sound memories (Osaki et al., 2005).

The increase in PTA thresholds are likely due to peripheral hearing loss and cochlear pathologies; however these change may contribute to central auditory disturbance that can contribute to presbycusis (Willott et al., 1993; Vasama and Mäkelä, 1997; Kraus et al., 2010; Newman et al., 2015). Impairments in peripheral hearing and central auditory processing have been linked to accelerated cognitive decline, incident cognitive impairment and AD (Gates and Mills, 2005; Panza et al., 2015). In elderly, non-demented subjects, Alzheimer neurofibrillary tangles are most often seen in the hippocampus and entorhinal cortex. However, in some cases they neurofibrillary tangles were also observed in BA 38 of the STG, suggestive of subclinical Alzheimer pathology (Vermersch et al., 1995).

fMRI has been used to detect the relationship between brain function in older adults during sentence processing and hearing ability; differences in hearing ability could predict the degree of neural recruitment in the bilateral STG during sentence comprehension (Peelle et al., 2011). Pink noise stimulation during fMRI imaging was used to examine the neural response in mild presbycusis patients and normal-hearing young controls (Profant et al., 2015). Presbycusis patients showed greater response to acoustical stimuli in the STG region compared with young subjects, indicative of sound evoked hyperactivity. Others used fMRI to explore the effect of hearing loss on engagement of the auditory cortex during the processing of monosyllabic words in presbycusis (Chen et al., 2016). Compared with age-matched healthy controls, bilateral activation in the auditory cortex was reduced in the presbycusis group. One MRS study also found reduced GABA concentrations in auditory regions of presbycusis patients and suggested that loss of GABA-medicated inhibition could contribute significantly to the deterioration of hearing function in the elderly (Gao et al., 2015). Our study extends these earlier reports by showing that presbycusis decreases the amplitude and local synchrony of spontaneous activity in the left STG (BA 38), which lies outside the primary auditory cortex. It is unclear if these decreases are mainly due to cochlear hearing loss, central auditory dysfunction or some combination of the two. Surprisingly, we did not observe a change in ALFF or ReHo in primary auditory cortex (BA 41, 42), regions in which electrophysiological studies in animal models showed increased spontaneous activity and neural synchrony with presbycusis or cochlear hearing loss degraded (Seki and Eggermont, 2003; Hughes et al., 2010; Chi-Wing and Recanzone, 2017). The reason for the difference between fMRI measures in humans and electrophysiological measure in primary auditory cortex of animals are clear, but could be due to methodological differences (e.g., anesthesia), species differences or duration of hearing loss or layer(s) of the cortex being evaluated (Novák et al., 2016).

In presbycusis patients, increased spontaneous activity and local synchrony in the MFG were associated with impaired cognitive performance on the TMT-B test, which assesses cognitive impairment related to executive function and damage to the frontal lobe (Tombaugh, 2004). The prefrontal cortex is mainly responsible for executive and cognitive functions (Roberts et al., 1998). Increased neuronal activity in the MFG may therefore have contributed to patients’ poor performance of the TMT-B test, which has been used to evaluate the executive dysfunction in central presbycusis (Gates et al., 2010). Husain et al. (2014) also observed increased connectivity between left MFG and seed region for the DMN in middle-aged patients with hearing loss. The aberrant spontaneous activity in the MFG play a pivotal role in presbycusis-related cognitive dysfunction related to executive function.

In our study, significant hypoactivity was found in subdivision of the DMN such as precuneus and IPL. The DMN, consisting of nodes in the precuneus/PCC, bilateral IPL, medial temporal gyrus and medial prefrontal gyrus, is most active at rest and shows reduced activity when a subject enters a task-based state involving attention or goal-directed behavior (Raichle et al., 2001; Mantini et al., 2007). Alterations to resting-state functional connectivity within the DMN have been detected in age-related hearing loss (Husain et al., 2014). The source or type of aberrant neural activity within specific DMN regions due to presbycusis remains unclear. Our results suggest that decreased ALFF/ReHo activity in precuneus and IPL may be responsible for disrupting the DMN in presbycusis patients.

Structural and functional alterations have been observed in the limbic system of presbycusis patients (Husain et al., 2014; Lin et al., 2014; Mudar and Husain, 2016). We observed a decrease in spontaneous activity and local connectivity in the right PHG, a primary node of the limbic system that plays a central role in memory encoding and recognition of environmental scenes and transferring information from the hippocampus to association areas (Diederen et al., 2010). Lin et al. reported atrophy of the PHG in older adults with hearing impairment compared with those with normal hearing (Lin et al., 2014). Moreover, Husain et al. (2014) used task-fMRI to demonstrate that positively and negatively valent sounds caused greater engagement of the limbic system compared to neutral sounds in normal-hearing individuals whereas older adults with hearing loss exhibited a decreased response in the PHG. Our results presumably indicate that these changes may be linked to disrupted resting-state neuronal activity in limbic system due to presbycusis.

The decline in ALFF/ReHo values in the STG, an auditory area, was associated with a complementary increase in the cuneus, a visual processing area and the PoCG, a somesthesis processing region. Decreased spontaneous activity in the auditory cortex due to hearing loss may promote spontaneous activity and/or cross-modal plasticity in visual and somatic processing areas consistent with other sound-activated imaging studies (Hadjikhani and Roland, 1998; Sadato et al., 2002; Zhang et al., 2006). A recent study utilizing rs-fMRI found that hearing loss modulated cross-modal functional connectivity between visual and auditory sensory cortices in presbycusis (Puschmann and Thiel, 2017). Auditory functional reorganization partly compensates for the needs of the visual system. Several studies observed enhanced visual and somatosensory projections into the auditory cortex, indicating that cross-modal plasticity as potential compensatory mechanism for presbycusis and/or hearing loss (Kok et al., 2014; Wong et al., 2015). Functional alterations observed in other sensory areas plus limbic and cognitive suggest that presbycusis is not limited to the classical auditory pathway, but involves sharing of multiple neurocognitive resources (Wayne and Johnsrude, 2015).

Since age-related hearing loss has been associated with structural changes, we compared GM and WM volumes but did not detect any differences between our presbycusis patients and matched controls. Previous studies have reported decreased GM volume associated with age-related hearing impairment in the central auditory and cerebral system (Peelle et al., 2011; Eckert et al., 2012; Lin et al., 2014). However, consistent with our results, Profant et al. reported no association between age-related hearing loss and GM volume (Profant et al., 2014). It is possible that the inherent heterogeneity of the hearing loss population may be one reason for the different results. Moreover, the MR technique and analytical method may also contribute to the differences.

Several limitations must be acknowledged in the current study. First, our cross-sectional study involved a relatively small sample size. Thus, it is difficult to make direct causal inferences regarding the relationships between the aberrant ALFF/ReHo and age-associated hearing loss. Further longitudinal fMRI studies are needed to establish to confirm the current findings. Second, we attempted to exclude subjects with tinnitus or hyperacusis from our study because subjects with tinnitus or hyperacusis exhibited resting-state brain abnormalities in auditory and non-auditory regions (Gu et al., 2010; Chen et al., 2017). However, it would be useful to include patients with tinnitus and hyperacusis in further studies in order to determine if spontaneous neural activity is disrupted in a similar manner to that observed in the current study. Finally, although we attempted to minimize the scanner noise with earplugs, we cannot rule out the possibility that scanner noise affected our results to some degree. This confounding factor should be taken into consideration in future studies.

## Conclusions

In this study, our combined ALFF and ReHo analyses demonstrated disrupted spontaneous neural activity in specific brain regions in presbycusis patients in the absence of any major structural changes. Aberrant spontaneous neural activity mainly occurred in the auditory cortex, prefrontal cortex and some parts of the DMN. Age-related hearing loss was associated with a decline in spontaneous activity of the auditory cortex whereas impaired cognitive/executive function was associated with increased spontaneous activity in the prefrontal cortex.

## Author Contributions

Y-CC designed the experiment, collected the data, performed the analysis and wrote the manuscript. HC, LJ, FB, J-JX, C-NM and XY collected the data. RS, GL and J-PG contributed to the discussion and manuscript revision.

## Conflict of Interest Statement

The authors declare that the research was conducted in the absence of any commercial or financial relationships that could be construed as a potential conflict of interest.

## Footnotes

^1^http://www.fil.ion.ucl.ac.uk/spm/software/spm8/

^2^http://www.restfmri.net
